# Diagnostic Efficacy of Chest Computed Tomography with a Dual-Reviewer Approach in Patients Diagnosed with Pneumonia Secondary to Severe Acute Respiratory Syndrome Coronavirus 2

**DOI:** 10.3390/tomography9050129

**Published:** 2023-08-24

**Authors:** Jaime E. Castellanos-Bermejo, Gabino Cervantes-Guevara, Enrique Cervantes-Pérez, Guillermo A. Cervantes-Cardona, Sol Ramírez-Ochoa, Clotilde Fuentes-Orozco, Gonzalo Delgado-Hernández, Jaime A. Tavares-Ortega, Erika Gómez-Mejía, Jonathan M. Chejfec-Ciociano, Juan A. Flores-Prado, Francisco J. Barbosa-Camacho, Alejandro González-Ojeda

**Affiliations:** 1 Departamento de Radiología e Imagen, Hospital General Regional 110, Instituto Mexicano del Seguro Social, Guadalajara 44716, Mexico; jaime.castellanos3448@alumnos.udg.mx; 2 Departamento de Bienestar y Desarrollo Sustentable, Centro Universitario del Norte, Universidad de Guadalajara, Colotlán 46200, Mexico; gabino_guevara@hotmail.com; 3 Departamento de Gastroenterología, Hospital Civil de Guadalajara Fray Antonio Alcalde, Universidad de Guadalajara, Guadalajara 44280, Mexico; 4 Departamento de Medicina Interna, Hospital Civil de Guadalajara Fray Antonio Alcalde, Guadalajara 44280, Mexico; enrique19896@msn.com (E.C.-P.);; 5 Centro Universitario de Tlajomulco, Universidad de Guadalajara, Tlajomulco de Zúñiga 45641, Mexico; 6 Departamento de Disciplinas Filosóficas, Metodológicas e Instrumentales, Centro Universitario de Ciencias de la Salud, Universidad de Guadalajara, Guadalajara 44340, Mexico; gacervantes66@hotmail.com; 7 Unidad de Investigación Biomédica 02, Unidad Médica de alta especialidad, Hospital de Especialidades Centro Médico Nacional de Occidente, Instituto Mexicano del Seguro Social, Guadalajara 44329, Mexico; clotilde.fuentes@gmail.com (C.F.-O.); medicinagon@gmail.com (G.D.-H.); albertotavares0610@gmail.com (J.A.T.-O.); erikagomejia@gmail.com (E.G.-M.); jchejfec@live.com (J.M.C.-C.); juanafp00@gmail.com (J.A.F.-P.); 8 Departamento de Psiquiatría, Hospital Civil de Guadalajara Fray Antonio Alcalde, Centro Universitario de Ciencias de la Salud, Universidad de Guadalajara, Guadalajara 44280, Mexico; efebeka_@hotmail.com

**Keywords:** COVID-19, pandemic, SARS-CoV-2, pneumonia, chest tomography, RT–PCR

## Abstract

To compare the diagnostic effectiveness of chest computed tomography (CT) utilizing a single- versus a dual-reviewer approach in patients with pneumonia secondary to severe acute respiratory syndrome coronavirus 2 (SARS-CoV-2), we conducted a retrospective observational study of data from a cross-section of 4809 patients with probable SARS-CoV-2 from March to November 2020. All patients had a CT radiological report and reverse-transcription polymerase chain reaction (PCR) results. A dual-reviewer approach was applied to two groups while conducting a comparative examination of the data. Reviewer 1 reported 108 patients negative and 374 patients positive for coronavirus disease 2019 (COVID-19) in group A, and 266 negative and 142 positive in group B. Reviewer 2 reported 150 patients negative and 332 patients positive for COVID-19 in group A, and 277 negative and 131 positive in group B. The consensus result reported 87 patients negative and 395 positive for COVID-19 in group A and 274 negative and 134 positive in group B. These findings suggest that a dual-reviewer approach improves chest CT diagnosis compared to a conventional single-reviewer approach.

## 1. Introduction

On 30 December 2019, a report published in Program for Monitoring Emerging Diseases (ProMED)-mail (program of the International Society for Infectious Diseases (ISID) staffed by a multidisciplinary global team of ISID Staff and over 50 subject matter experts reporting from 34 countries), indicated the presence of patients with pneumonia of unknown etiology in Wuhan, China [1,2]. After initial investigations to identify the causative agent, the World Health Organization named it the 2019 novel coronavirus [1]. The most common symptoms are fever, dry cough, fatigue, headache, chest pain, odynophagia, and myalgia. In addition, gastrointestinal symptoms, including abdominal pain and diarrhea, have been reported [3,4,5]. Reverse-transcription polymerase chain reaction (RT–PCR) is the diagnostic criterion standard [6]. Tests performed during the Wuhan outbreak have shown various degrees of sensitivity, ranging from 37% to 71% [7], in which deficiencies such as inadequate collection, manual errors, or contamination of samples are noted [8]. Chest computed tomography (CT) is one of the methods used in diagnosing coronavirus disease 2019 (COVID-19) [9], and as knowledge about pneumonia secondary to severe acute respiratory syndrome coronavirus 2 (SARS-CoV-2) became more formalized, protocols for both management and diagnosis appeared. The COVID-19 Reporting and Data System (CO-RADS) for chest CT interpretation was accepted to organize and structure the information for better performance in patients with pneumonia secondary to SARS-CoV-2 infection [10]. In Wuhan, 1014 patients underwent chest CT interpreted and endorsed by two radiologists through a separate review, which was then merged to produce a single result and RT–PCR for SARS-CoV-2. This study employed the RT–PCR test as the reference standard and chest CT for diagnosing COVID-19-related pneumonia. When compared directly, chest CT had a sensitivity of 97% [11]. An interesting question about chest CT and RT–PCR testing is whether chest CT has similar or better diagnostic performance than RT–PCR testing in the initial assessment [12].

Given this background, the present study aimed to quantify the diagnostic efficacy of chest CT using a dual-reviewer approach in patients with SARS-CoV-2-associated pneumonia based on sensitivity, specificity, positive predictive value (PPV), and negative predictive value (NPV) as a replacement for RT–PCR.

In their review of the diagnostic tools for COVID-19, Alsharif and Qurash [13] established low sensitivity for RT–PCR but high sensitivity for initial chest CT, making it possible to correct false-positive RT–PCR test results, which also suggested that CT has low specificity and therefore requires solving the latter to improve the diagnostic yield. They also argued that artificial intelligence (AI) could increase the ability to differentiate COVID-19 from other types of viral pneumonia, supporting the idea that chest CT alone requires complementary strategies or additional techniques to increase its diagnostic yield, and given that in our environment, there is no appropriate AI software, proposing a methodological change different from the conventional one (a single reviewer), even when it seems obvious, requires measuring if a complementary methodology (dual reviewers) increases the diagnostic yield.

Reginelli et al. [14] stated that in discordant cases of COVID-19, the double reading of CT scans and second expert readers could increase the diagnostic confidence of radiological interpretation in patients with COVID-19. Fonseca et al. [15] reported the assessment by three radiologists with various experience. Their study focused on assessing the intraobserver and interobserver correlations, highlighting that the experience of radiologists influences the performance of imaging studies. In this case, the addition of a third reviewer allows consensus when discriminating indeterminate cases, making clear that it is necessary to have the most reliable information possible to improve diagnostic performance.

In 2020, Falaschi et al. [7] published an article in which the performance of chest CT was evaluated and compared with RT–PCR and showed better sensitivity and specificity than previously published studies, highlighting that its methodology is based on the fact that two radiologists with more than ten years of experience in thoracic imaging evaluated the images in consensus while being blinded to the RT–PCR results. We considered that although the article established this methodology, it is not the standard in the interpretation of imaging studies, and in Mexico, it is not a methodology conventionally disseminated.

## 2. Materials and Methods

We conducted a retrospective observational study of data from a cross-section of 4809 patients suspected of having SARS-CoV-2 from March to November 2020, of whom 3193 were confirmed to be positive for the disease by RT–PCR; the remaining 1616 were excluded. The patients were classified into two groups. The inclusion criterion for group A was RT–PCR (positive) for SARS-CoV-2 in a chest CT radiological report. As a result, 482 patients were included in the analysis. The inclusion criterion for group B was RT–PCR (negative) for SARS-CoV-2 in a chest CT radiographic report. As a result, 408 patients were included in the analysis.

The exclusion criteria were those who did not meet the inclusion criteria and those whose imaging studies could not determine whether they were positive or negative for COVID-19-associated pneumonia according to their imaging characteristics.

We performed nonprobabilistic quota sampling (RT–PCR results) to integrate the two groups of patients according to the sample size specifications. The imaging reports for each patient and the RT–PCR test results were compiled to integrate the patient data and the results of both tests into a data collection sheet designed in Excel 2017 Microsoft Corporation. The following finite population formula was used for the representative sample:

$$n=Z^{2}\times P\times Q\times {\frac{N}{E}}^{2}\times \left(N-1\right)+Z^{2}\times P\times Q$$

where *n* is the sample size, *N* is the size of the study population, *P* and *Q* are the probability of the phenomena studied, *Z* is the critical value associated with the chosen confidence level, and *E* is the sampling error.

The diagnostic efficacy of chest CT was compared with the RT–PCR test results, which is considered the criterion standard for SARS-CoV-2-associated pneumonia. The radiological reports were made by imaging specialists blinded to the patient’s diagnosis. Subsequently, a dual-reviewer approach was applied to each of the tomography studies, in which the radiologists were blinded to the previous report for each patient and the result of the RT–PCR test. After the second review of the CT, the results of the imaging studies were compiled, whereby both radiologists interpreted the findings and entered them into a table designed in Excel. Based on these findings, the sensitivity, specificity, PPV, and NPV were calculated. Finally, we compared the data for each item obtained, that is, sensitivity, specificity, PPV, and NPV, to determine whether the dual-reviewer approach was more effective than the single-reviewer approach. The kappa was interpreted as fair (>0.20), moderate (>0.40), substantial (>0.60), and almost perfect agreement (>0.80) [16].

As knowledge of SARS-CoV-2-associated pneumonia became more formalized, proposals for diagnostic and management protocols began to appear in the scientific literature. Thus, the CO-RADS reporting system was adopted to interpret chest CT to organize and structure the information and generate a clear and internationally comprehensible language for the diagnosis of COVID-19. This system was developed by the Dutch Society of Radiology and is based on the existence of other similar systems for diagnosis and report generation in other pathologies, including the Lung Imaging Reporting and Data Reporting System (LI-RADS), the Prostate Imaging Reporting and Data Reporting System (PI-RADS), and the Breast Imaging Reporting and Data Reporting System (BI-RADS), achieving consensus that this structuring of imaging reports and reports is effective [10].

Patients were classified based on the CO-RADS scale as follows.

CO-RADS 0: indeterminate; inability to designate a value based on an unintelligible or technically inadequate study.

**Figure 1:**
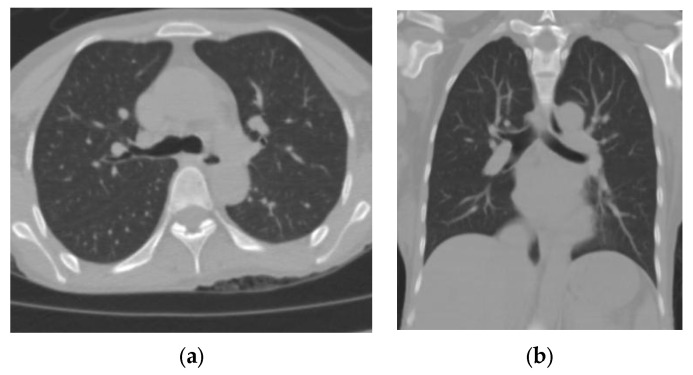
(**a**) Axial section and (**b**) coronal section of a patient who underwent an RT–PCR test for SARS-CoV-2 upon admission. The finding was negative. He was discharged after improvement at 3 weeks following admission.

CO-RADS 1: negative; normal examination or findings not associated with a pulmonary infection (Figure 1).

**Figure 2:**
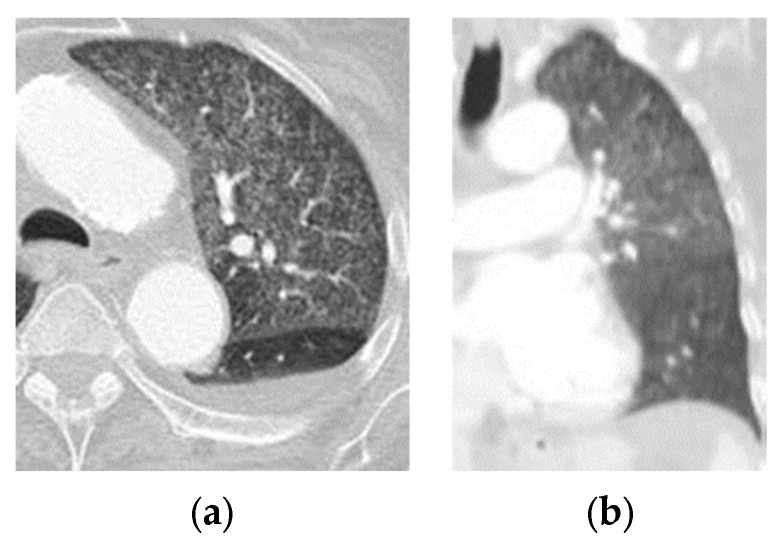
(**a**) Axial section and (**b**) coronal section of a patient with dyspnea and negative test for RT-PCR for SARS-CoV-2. Thorax window lung where the miliary pattern of TB is confirmed.

CO-RADS 2: negative; study with typical findings of infections other than COVID-19 (Figure 2).

**Figure 3:**
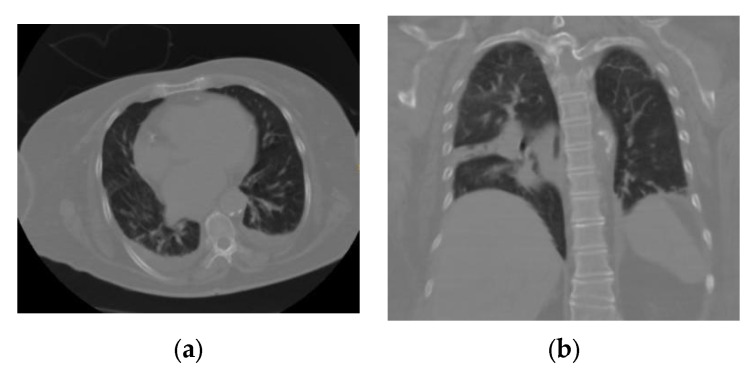
(**a**) Axial section and (**b**) coronal section of a patient with an indeterminate interstitial opacity pattern that, being unique, septal, and lobular with associated pleural effusion, did not allow confirmation or rule out an association with COVID-19; these findings may be associated with any viral pneumonia (in this case, the patient had a negative RT–PCR test result for SARS-CoV-2).

CO-RADS 3: a study with uncertain or indeterminate findings that shows areas suggestive of COVID-19, but also of other pathologies (Figure 3).

**Figure 4:**
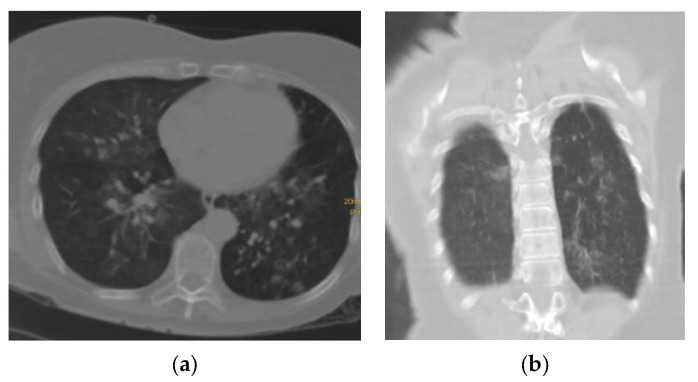
(**a**) Axial section and (**b**) coronal section of a patient with a pattern of multifocal interstitial ground-glass opacity characteristic of pneumonia associated with COVID-19 (SARS-CoV-2); however, it is accompanied by fine bands of lamellar atelectasis bilaterally without frank consolidation, which may suggest preexisting lung disease.

CO-RADS 4: positive; study with suspicious findings of COVID-19 with superimposition of some findings not typical of other pathologies (Figure 4).

**Figure 5:**
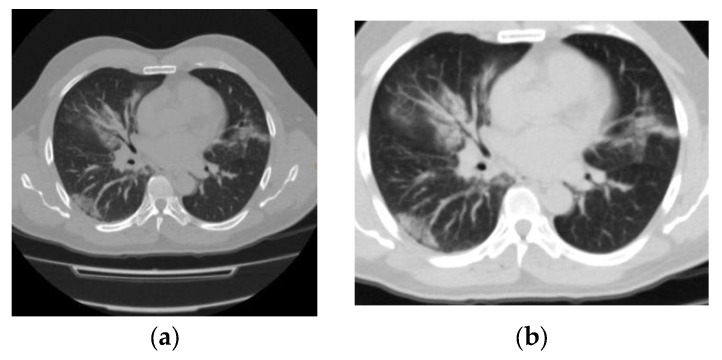
(**a**) Axial section and (**b**) axial section (with magnification) show a pattern of multifocal interstitial ground-glass opacity characteristic of pneumonia associated with COVID-19 (SARS-CoV-2) and highly suggestive of the disease in this patient. She was admitted to the intensive care unit after chest CT, showed a positive RT–PCR test result 48 h later, and died 7 days after admission.

CO-RADS 5: positive; study with typical findings of COVID-19 (Figure 5).

CO-RADS 6: proven; patient RT–PCR positive for SARS-CoV-2. This category has no characteristic image as it represents patients already known to be diagnosed by RT-PCR test; therefore, no image is included.

**Figure 6:**
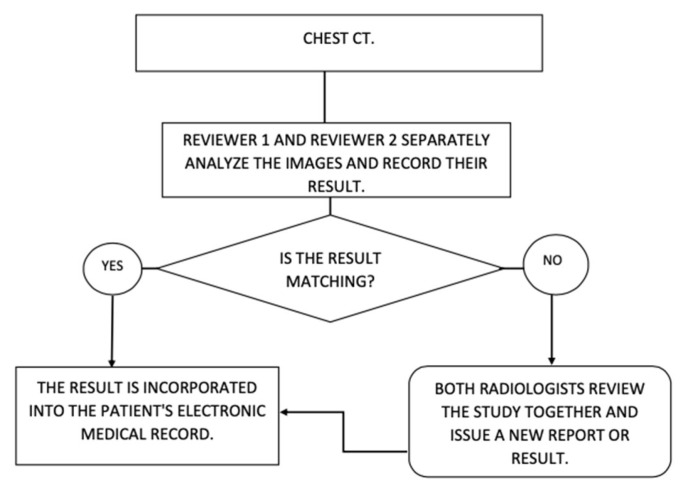
Result of each reviewer and consensus.

How the results of each reviewer and the consensus will be obtained will be as follows: each one of the reviewers will separately assess each of the cases and record their result with one of the CO-RADS categories, if applicable. If they coincide in the same category, it is taken as consensus; if they do not coincide, both analyze the study together and issue a single result in a CO-RADS category (Figure 6).

## 3. Results

We included data from 890 patients with a mean age of 57.9 ± 19.07 years, of whom 366 (41.1%) were women, 524 (58.9%) were men, 482 (54.2%) were COVID-19-positive, and 408 were COVID-19-negative according to an RT–PCR test for SARS-CoV-2. The patients had an average duration of hospital stay of 7.45 ± 8.22 days (range, 0–58 days). The average duration between hospital admission and patient death was 18.88 ± 31.81 days (1–259 days). The clinical classification criteria were as follows: 192 (21.6%) were respiratory tract infections, and 698 (78.4%) were acute respiratory tract infections. The CO-RADS score reported by reviewer 1 was, on average, higher than that reported by reviewer 2; this score was classified as equal in 533 (59.9%) of the cases (Table 1).

To obtain the performance of individual reviewers and the dual-reviewer approach, sensitivity, specificity, PPV, and NPV were obtained using 2 × 2 tables and applying the formulas for each case (Table 1 and Table 2). In all cases, including the consensus approach, the CT studies found that CO-RADS scores 4 and 5 were considered positive for SARS-CoV-2 (COVID-19). Likewise, CO-RADS scores 1–3 were considered negative.

Reviewer 1 reported 108 patients negative and 374 patients positive for COVID-19 in group A (positive RT–PCR test results for SARS-CoV-2) and 266 negative and 142 positive in group B (negative RT–PCR test results for SARS-CoV-2). Reviewer 2 reported 150 patients negative and 332 positive for COVID-19 in group A and 277 negative and 131 positive in group B (Table 3).

The consensus result reported 87 patients negative and 395 positive for COVID-19 in group A (positive RT–PCR test results for SARS-CoV-2), and 274 negative and 134 positive in group B (negative RT–PCR test results for SARS-CoV-2) (Table 4). The kappa index obtained was 0.64 for CO-RADS 4–5, very suspicious, versus CO-RADS 1–3, nonsuspicious, indicating good agreement between the two examiners.

**Figure 7:**
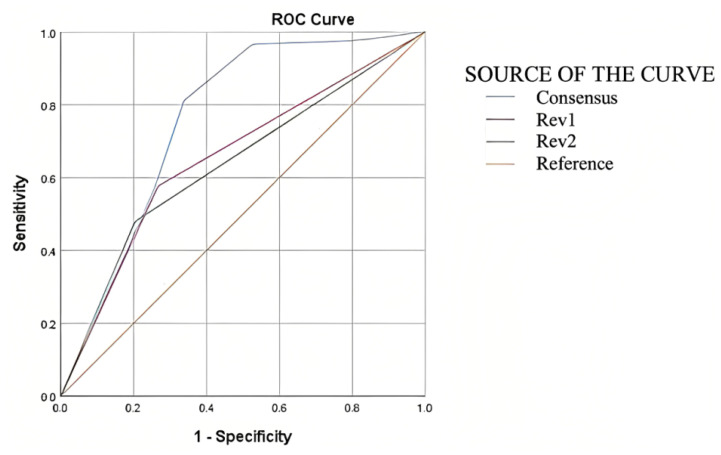
Area under the ROC curve for both reviewers and consensus.

We evaluated the findings of the chest CT diagnostic tests using a receiver operating characteristic (ROC) curve analysis to determine the diagnostic accuracy of this test in such a way that although the findings of both reviewers were similar, the sensitivity and specificity were obtained separately within the expected ranges (Table 4). Thus, we found evidence of a notable increase in the accuracy of chest CT when the dual-reviewer approach was applied through consensus (Table 5 and Figure 7).

The advantages of the consensus can be explained by the indeterminate categories, which are optimized. Therefore, we can affirm that although chest CT in patients with SARS-CoV-2 (COVID-19) does not replace the value of an RT–PCR test for SARS-CoV-2 as a criterion standard, it should be considered a valuable test for decision-making in patients with this clinical condition. Furthermore, when applying the dual-reviewer approach, its value has statistical significance.

## 4. Discussion

In the present study, we found that the dual-reviewer approach was 6.5% more sensitive than the conventional single-reviewer approach, with 82% compared with 78% sensitivity for reviewer 1, 69% for reviewer 2, and 73.5% for the average.

Our hospital’s dual-reviewer approach boosted sensitivity by 6.5%, specificity by 2%, PPV by 3%, and NPV by 8% compared with the single-reviewer approach. Therefore, the dual-reviewer approach improved chest CT test performance, supporting the claim by Reginelli et al. [14] that a double reading and structured report improved the test’s diagnostic capacity, obtaining a sensitivity of 97.3%, specificity of 53.8%, PPV of 89%, and NPV of 88.4%. Those investigators concluded that the dual-reviewer approach could increase the diagnostic confidence of radiological interpretation in patients with COVID-19.

A review of 1014 patients who underwent chest CT and RT–PCR for SARS-CoV-2 in Wuhan, China, found that the sensitivity for CT was 97% [11]. According to that study’s methodological description, the dual-reviewer approach is an ideal diagnostic tool for initial situations such as those detailed in this research. In their conclusions, the authors noted that a variable to consider is the onset of symptoms relative to chest CT, the initial RT–PCR test results for SARS-CoV-2, and the possibility of additional RT–PCR tests, which were not performed in our setting [11].

A study from India that included 612 patients and compared chest CT to RT–PCR for SARS-CoV-2 found that chest CT had a sensitivity of 94.2%, 14.2% greater than the sensitivity found in the present study. The specificity of 76.4% was 9.4% greater than that found in the present study, and the PPV of 76.7% was 1.7% higher than we found. However, it should be noted that the number of patients in that study was smaller than that in the present study and that its primary objective was to compare chest CT with RT–PCR. Regardless, the diagnostic performance of chest CT was higher; therefore, additional factors that may affect the optimal performance of the diagnostic tests need to be considered [17]. A direct comparison with studies conducted abroad allows us to place the present study in the context of others conducted during the COVID-19 pandemic and those that, because of their sample size or methodology, are the most relevant in the review conducted by Kovács [18], which compiles the values of other studies and shows some variability between them, indicating that the present study has a similar sample size to that in the study by Ai et al. [11], who found lower sensitivity but greater specificity. That review indicates that the sensitivity and specificity in the present study are within the range of reports worldwide.

The sensitivity of CT was 97.2% at presentation, whereas that of the initial RT–PCR was 84.6% [19]. Owing to a shortage of kits and the false-negative rate of RT–PCR, clinicians in Hubei Province, China temporarily used chest CT to diagnose COVID-19. Chest CT has a higher sensitivity (86–98%) and a lower incidence of a false-negatives than RT–PCR. The main caveat of chest CT for COVID-19 is its low specificity (25%) because the imaging characteristics overlap with those of other viral pneumonia [20]. Asymptomatic patients with COVID-19 can show paradigmatic CT changes very early, even before being found positive according to RT–PCR. Therefore, clinicians may be unable to diagnose a patient with COVID-19 at an early stage because of a false-negative RT–PCR test result [21]. According to 37 studies, the sensitivity of CT compared with RT–PCR was 87% (95% CI: 85–90%), and according to seven studies, the specificity of CT was 46% (95% CI: 29–63%) [22]. In 20 studies using CO-RADS scores < 3, the pooled sensitivity and specificity were 0.89 (95% CI: 0.85–0.93) and 0.68 (95% CI: 0.60–0.75), respectively. In 23 studies using CO-RADS scores < 4, the pooled sensitivity and specificity were 0.83 (95% CI: 0.79–0.87) and 0.84 (95% CI: 0.78–0.88), respectively. In 21 studies using CO-RADS scores < 5, the pooled sensitivity and specificity were 0.66 (95% CI: 0.61–0.72) and 0.93 (95% CI: 0.88–0.96), respectively [23]. According to other investigators, the CO-RADS score is adequate for classification, diagnosis, management decisions, and prognosis. Almost all patients in the present study who required treatment in the intensive care unit had CO-RADS scores < 3, 115 (89.2%) of those transferred to the intensive care unit had CO-RADS scores < 4, and 36 (97.3%) of those who died because of SARS-CoV-2 infection had CO-RADS scores < 4. Even in the C-reactive protein (CRP)-negative group, we observed that approximately half of the CRP-negative patients (*n* = 210) had a CO-RADS score < 2. Among them, the number of patients with a score of 5 referring to severe lung disease was considerably higher [24]. Our present results show that using a CO-RADS score > 3 as the optimal diagnostic threshold, both highly and less experienced radiographers were able to identify patients with COVID-19, with mean areas under the curve of 0.72 (95% CI: 0.68–0.75) and 0.71 (95% CI: 0.68–0.74), respectively. Chest CT correctly identified 67 of 102 patients as positive for COVID-19-associated pneumonia. Therefore, an initial chest CT is reliable and valid for detecting COVID-19 rapidly, possibly modifying the management of the 66% of patients misidentified as negative by their initial RT–PCR test results [25]. Compared with the average performance of the conventional single-reviewer approach [26], the dual-reviewer approach used increased sensitivity by 6.5%, specificity by 2%h, PPV by 3%, and NPV by 8% in our hospital (Table 6).

Islam et al. [31] published a Cochrane review of 84 studies that did not report on the methodology with which the CT was reviewed. However, they reported heterogeneity in the data obtained and in the results of the interpretation of the same in such a way that it was not possible to establish whether a single- or dual-reviewer methodology is followed in most of the articles; this remains an information gap that is not resolved in most articles. Differences were found between the reviewers, making it necessary to improve the precision; the most extensive articles and the first studies, such as that published by Long et al. [20], clearly define that their review methodology is with two reviewers, as do Ai et al. [11] in the article with the most extensive number of patients published with 1014 patients, in which they establish that two radiologists evaluated chest CT findings, concluded a diagnostic yield with sensitivity close to 97%, and established that the improvement in CT is ahead of the negativization of RT–PCR. From the previous, it can be noted that the judgment of a single person reduces the value of the qualification of an image study; having an additional person review the images improves the analysis and the precision with which the images are evaluated (Figure 6).

## 5. Conclusions

The utilization of the dual-reviewer strategy yielded a sensitivity rate of 82% and a specificity rate of 67%. While the sensitivity observed in this study was comparatively lower than that reported in other research, it is worth noting that the specificity showed an increase. This improvement in specificity can be attributed to the utilization of a larger sample size, which allowed for a more representative analysis. In addition to reducing the subjectivity of the reviewers, the dual-reviewer approach enhanced the diagnostic performance of chest CT by 6.5% (sensitivity) and 8% (NPV) compared with the single-reviewer approach. When the dual-reviewer approach is used, the consensus result is significantly better and should be deemed a diagnostic test for COVID-19 (SARS-CoV-2), supporting the hypothesis that this approach improves test performance.

However, the main drawback of the proposed approach is that it requires two radiologists and imaging specialists to perform the same task, which some administrations may not be able to afford. Therefore, studies that analyze the cost–benefit ratio of having and dedicating these resources to patients with COVID-19 are recommended. In light of these findings, future research may examine the follow-up of patients with COVID-19-associated pneumonia and the diagnostic performance of RT–PCR tests for SARS-CoV-2 to determine their actual diagnostic value.

## Figures and Tables

**Table 1:** CO-RADS score category for each reviewer and consensus.

CO-RADS					
	1	2	3	4	5
Reviewer 1	94 (10.6%)	134 (15.1%)	146 (16.4%)	129 (14.5%)	387 (43.5%)
Reviewer 2	104 (11.7%)	97 (10.9%)	226 (25.4%)	149 (16.7%)	314 (35.6%)
Consensus	93 (10.4%)	116 (13%)	152 (17.1%)	153 (17.2%)	376 (42.2%)

**Table 2:** Category of CO-RADS scores from each reviewer and consensus according to the RT–PCR test results for SARS-CoV-2.

RT–PCR SARS-CoV-2			
	CO-RADS	Positive (A)	Negative (B)
Reviewer 1	1	12 (2.5%)	82 (20.1%)
	2	8 (1.7%)	126 (30.9%)
	3	88 (18.3%)	58 (14.2%)
	4	96 (19.9%)	33 (8.1%)
	5	278 (57.7%)	109 (26.7%)
Reviewer 2	1	17 (3.5%)	87 (21.3%)
	2	17 (3.5%)	80 (19.6%)
	3	116 (24.1%)	110 (27%)
	4	101 (21%)	48 (11.8%)
	5	231 (47.9%)	83 (20.3%)
Consensus	1	11 (2.2%)	82 (20.1%)
	2	4 (0.9%)	112 (27.4%)
	3	72 (14.9%)	80 (19.6%)
	4	122 (25.3%)	31 (7.6%)
	5	273 (56.6%)	103 (25.2%)

**Table 3:** Results for each reviewer and consensus.

RT–PCR SARS-CoV-2 Chest CT	Positive for SARS-CoV-2	Negative for SARS-CoV-2
Reviewer 1		
Positive for COVID-19	374	142
Negative for COVID-19	108	266
Reviewer 2		
Positive for COVID-19	332	131
Negative for COVID-19	150	277
Consensus		
Positive for COVID-19	395	134
Negative for COVID-19	87	274

SARS-CoV-2, severe acute respiratory syndrome coronavirus 2; COVID-19, coronavirus disease 2019.

**Table 4:** Diagnostic performance results for each reviewer and consensus.

	Sensitivity	Specificity	Positive Predictive Value	Negative Predictive Value
Reviewer 1	78%	65%	72%	71%
Reviewer 2	69%	68%	72%	65%
Consensus	82%	67%	75%	76%

**Table 5:** Area under the ROC curve for both reviewers and consensus.

Area under the ROC Curve					
Test Result Variable(s)	Area	Std. Error	Asymptotic Sig.	Asymptotic 95% Confidence Interval	
			*p*	Lower Bound	Upper Bound
Reviewer 1	0.655	0.018	<0.001	0.619	0.691
Reviewer 2	0.638	0.019	<0.001	0.602	0.674
Consensus	0.760	0.017	<0.001	0.726	0.798

**Table 6:** Comparison with other studies.

Article	Sensitivity	Specificity	Sample Size
Ai et al. [11]	97%	25%	1014
Wu et al. [27]	95%	Not reported	80
Himoto et al. [28]	67–83%	80–93%	21
Cheng et al. [29]	100%	25%	38
Caruso et al. [30]	97%	56%	158
Present study	82%	67%	890

## Data Availability

The datasets used and/or analyzed during this study are available from the corresponding author upon reasonable request.
